# A Case of Coronary Sinus Atrial Septal Defect With Left Ventricular Thrombosis Treated With Minimally Invasive Cardiac Surgery

**DOI:** 10.7759/cureus.82260

**Published:** 2025-04-14

**Authors:** Yoshun Sai, Keita Kikuchi, Joji Ito

**Affiliations:** 1 Department of Cardiovascular Surgery, Tokyo Bay Urayasu Ichikawa Medical Center, Chiba, JPN; 2 Department of Cardiovascular Surgery, Tokyo Bay Urayasu Ichikawa Medical Center, chiba, JPN

**Keywords:** coronary sinus atrial septal defect, left ventricle thrombus, minimally invasive cardiac surgical procedures, septal defect patch closure, surgical thrombectomy

## Abstract

Coronary sinus atrial septal defect (CS‑ASD) is an uncommon congenital anomaly that accounts for <1% of all atrial septal defects. Over the past decade, around 10 adult CS‑ASD cases have been reported, and none have included simultaneous left ventricular (LV) thrombectomy.

We describe the case of a 71‑year‑old man who presented with chest pain and ST‑segment‑elevation myocardial infarction caused by proximal right coronary artery occlusion, which was successfully treated with stent placement. Subsequent cardiac examination revealed CS-ASD and LV thrombosis. Using an endoscopic approach, we successfully repaired the defect and removed the thrombus.

Because the defect lacked an adequate surrounding rim and a transcatheter device could jeopardize coronary‑sinus patency, percutaneous closure was deemed contraindicated, and thus surgical patch repair was undertaken. Endoscopic surgery performed via a right mini‑thoracotomy afforded excellent exposure of the atrial septum and LV cavity while being less invasive and allowing sternal preservation, thereby facilitating an expedited postoperative recovery, advantages that are particularly pertinent when concomitant CS‑ASD closure and LV thrombectomy are required.

## Introduction

Coronary sinus atrial septal defect (CS‑ASD), a communication between the coronary sinus (CS) and the left atrium (LA), is classified as a variant of unroofed coronary sinus (URCS) syndrome, which itself belongs to the broader spectrum of atrial septal defects (ASDs). This rare congenital anomaly accounts for less than 1% of all ASDs [1]. URCS comprises a continuum of defects in which part or all of the common wall between the CS and the LA is absent, and it may or may not be associated with a persistent left superior vena cava (PLSVC). The most severe form involves a completely unroofed CS with direct drainage of the CS and PLSVC into the LA, leading to a right‑to‑left shunt and paradoxical embolism [1,2].

Because clinical manifestations are often subtle, establishing the diagnosis can be challenging. Transthoracic echocardiography (TTE) may miss the defect owing to its posterior location and acoustic shadowing; therefore, transesophageal echocardiography, cardiac computed tomography (CT), or magnetic resonance imaging are frequently required for definitive visualization of the CS roof and associated shunting. As a result, CS‑ASD is frequently detected only in adulthood, when patients may present with dyspnea, arrhythmias, or paradoxical embolic events.

Surgical correction of CS‑ASD typically involves patch closure of the defect via median sternotomy and cardiopulmonary bypass; however, thoracoscopic minimally invasive cardiac surgery (MICS) can shorten recovery and reduce wound‑related complications because of its smaller incisions, yet it remains uncommon due to the rarity of CS‑ASD.

Moreover, concurrent pathologies such as left ventricular (LV) thrombus - an uncommon but potentially life‑threatening condition - pose additional challenges. These challenges include the risk of systemic embolic events such as ischemic stroke, renal or splenic infarction, and acute limb ischemia. Although the prevalence of LV thrombus has declined with advances in guideline‑directed anticoagulation, cases that do not respond adequately to anticoagulation carry a high risk of systemic embolization, for which surgical removal is recommended.

Here, we present a rare case of CS‑ASD complicated by an LV thrombus, in which simultaneous thoracoscopic minimally invasive surgical repair of the CS‑ASD and removal of the LV thrombus was successfully accomplished. Our experience suggests that MICS may evolve into a less‑invasive standard approach for selected adult patients with CS‑ASD, even in the presence of complex concomitant intracardiac pathology.

## Case presentation

**Figure 1:**
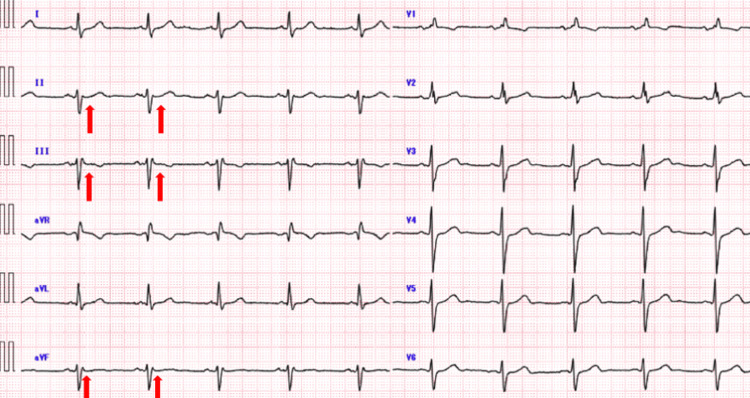
Preoperative electrocardiography. Electrocardiography revealed sinus rhythm with no ST-T changes in the inferior leads (II, III, and aVF) (red arrows).

A 71-year-old man presented to his local emergency department approximately 2 hours after the sudden onset of substernal chest pain. Electrocardiography revealed ST-segment elevation in the inferior leads (II, III, and aVF), and serum troponin I levels were elevated, consistent with an acute inferior ST-elevation myocardial infarction (STEMI). Emergency coronary angiography demonstrated an occlusion of the proximal right coronary artery, and primary percutaneous coronary intervention was performed at the referring hospital. TTE performed immediately after the intervention revealed what was initially interpreted as a secundum‑type ASD together with a mobile thrombus attached to the inferior LV wall. Warfarin anticoagulation was initiated in accordance with the American Heart Association (AHA) guideline recommendations, and the patient was referred to our institution for definitive management. The patient had no heart murmur or edema of the extremities. Electrocardiography revealed sinus rhythm with no ST-T changes in the inferior leads (II, III, and aVF) (Figure 1).

**Figure 2:**
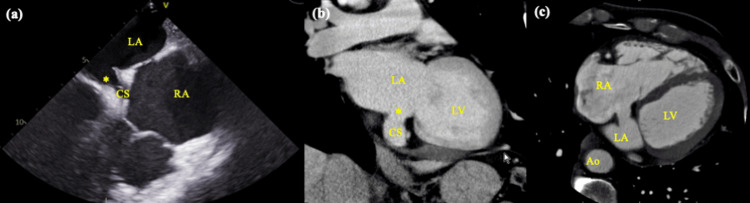
Preoperative findings. (a) Transesophageal echocardiography showing a defect along the roof of the coronary sinus, creating a direct communication between the left and right atria, consistent with coronary sinus-type atrial septal defect (CS-ASD). (b, c) Contrast-enhanced CT imaging showing a discontinuity in the wall of the coronary sinus, indicating the presence of a septal defect opening into the CS, as well as a right aortic arch. Ao, aorta; CS, coronary sinus; LA, left atrium; LV, left ventricle; RA, right atrium *Unroofed portion of the coronary sinus

TTE showed reduced LV function with an ejection fraction of 37%, an ASD (Qp/Qs: 1.9), trivial tricuspid regurgitation, and a 10mm-sized mass suspected of thrombus within the inferior LV wall, which remained unchanged in size despite anticoagulation therapy for three months. TEE showed an 8.1 × 13.2 mm coronary sinus type ASD (CS‑ASD) rather than a secundum defect (Figure 2a). CT showed a right aortic arch and an ASD opening into the CS (Figures 2b, 2c) but no PLSVC. Despite three months of anticoagulant therapy, there was no change in the size of the LV thrombus. Given the risk of systemic embolization, we elected to proceed with surgical intervention. ASD closure was also planned not only because Qp/Qs exceeded 1.5 but also to eliminate the potential for paradoxical embolism and progressive right heart volume loading in the setting of impaired LV function.

**Figure 3:**
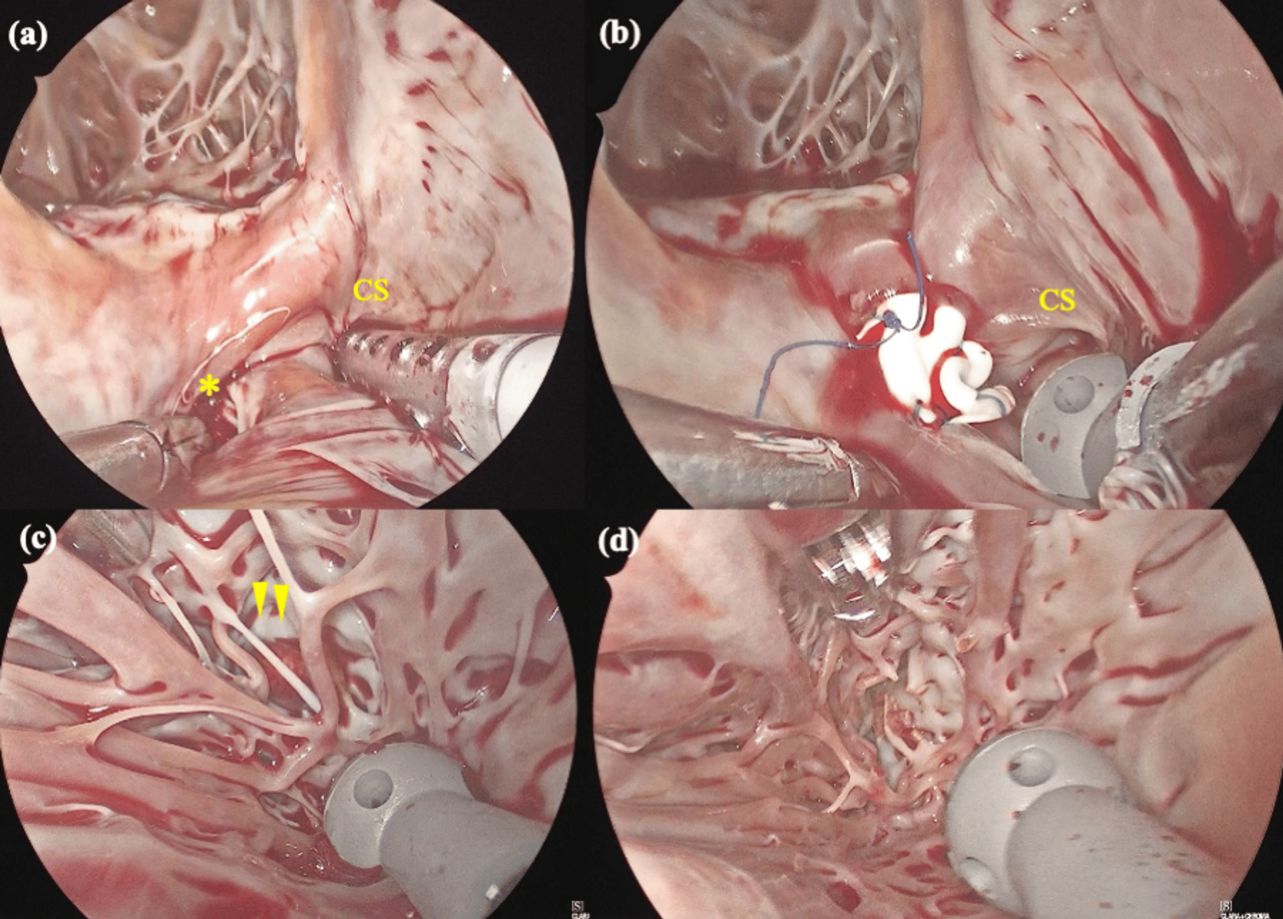
Intraoperative findings. (a) Atrial septal defect opening into the CS was observed (1cm x 1cm). (b) Defect was closed by the Gore-Tex patch. (c) The thrombus was partially embedded within the LV trabeculae carneae, with its base adherent to the endocardial surface. (d) Completion of the removal with resection a part of trabeculae. CS, coronary sinus *Unroofed portion of the CS. Arrowheads indicates thrombosis.

A lateral right mini-thoracotomy was performed via the fourth intercostal space. Cardiopulmonary bypass (CPB) was established via the right femoral artery and vein with additional drainage through the right internal jugular vein, a strategy that facilitates minimally invasive access and obviates direct aortic cannulation in the narrow operative field. After antegrade cardioplegia, the right atrium was opened. The ASD was exposed through the right atriotomy, which opened into the CS, as shown in the preoperative echocardiographic findings (Figure 3a). A 10 × 10 mm defect communicating with the CS was observed. Remnant septum was present, and thus a Gore‑Tex patch was used to reconstruct the wall between the LA and the CS. The patch was secured using a continuous 5‑0 polypropylene suture, effectively rerouting the CS drainage into the right atrium and closing the interatrial communication (Figure 3b). Subsequently, the LA was then opened at Waterson’s groove, an approach that provides a direct line of sight to the LV cavity through the mitral valve. In the left ventricle, a thrombus was partially embedded within the LV trabeculae carneae, with its base adherent to the endocardial surface (Figure 3c). The mass was removed en bloc using gentle suction and intermittent warm saline irrigation to prevent fragmentation and systemic embolization; a small segment of trabeculae was resected to free its base (Figure 3d).

The patient was weaned off CPB without complications. The aortic cross-clamp time was 133 minutes, and the total CPB time was 195 minutes. Early postoperative TTE demonstrated unchanged LVEF, with no residual inter‑atrial shunt and no LV thrombus. The patient was discharged on postoperative day 7 with no conduction disturbances. In accordance with current AHA guidelines favoring vitamin K antagonists for post‑myocardial infarction LV thrombus, warfarin anticoagulation was therefore continued for a planned six‑month period due to residual LV dysfunction. At six months, TTE follow‑up confirmed no residual or recurrent LV thrombus, and the patient remained asymptomatic. The duration of anticoagulation will be further re‑evaluated based on ongoing serial imaging.

## Discussion

CS-ASD is found in less than 1% of ASDs [1]. There are four types of uncovered CS syndrome, based on the presence or absence of PLSVCs [2]. As there was only a defective hole at the terminal end of the CS and no association with PLSVC, the present case was diagnosed as type IV, a partially unroofed terminal segment. Type IV is clinically significant as it typically lacks a PLSVC and tends to present in adulthood with subtle symptoms, making diagnosis more challenging. Surgically, the defect is often small and well localized, which may allow for a more straightforward repair compared to types I-III, which are frequently associated with PLSVCs and require rerouting or more extensive reconstruction.

In this case, both transesophageal echocardiography (TEE) and contrast-enhanced CT were used to establish the diagnosis. The multimodal imaging was instrumental in confirming the presence and precise location of the CS-ASD and in ruling out other associated anomalies such as PLSVC or atrioventricular septal defects.

Patients with CS-ASD can be treated with percutaneous closure or surgery. Kijima et al. reported a successful case of CS-ASD percutaneous closure using an Amplatzer™ Septal Occluder (Abbott, Chicago, IL, USA) in 2012 [3]. However, the indications are limited owing to problems with the opening diameter and rim width, as well as concerns about erosion caused by the device. Specifically, device closure is often unfeasible when the CS-ASD has irregular or crescent-shaped margins, deficient rims, or a defect diameter greater than 10-12 mm, which exceeds the available occluder sizes.

Surgical treatment includes patch closure or direct suture closure; however, the former is preferred when the defect hole is large because of the risk of tears and damage to the atrioventricular node and bundle of His with direct suturing. Direct suturing is generally considered safe for defects smaller than 5 mm; for larger defects, patch closure is recommended to minimize tension and reduce conduction system injury.

In a systematic review and meta-analysis, the MICS approach was reported to be predominantly more effective in treatment and have fewer residual shunts than device closure [4]. Despite a longer hospital stay compared to device closure, the MICS approach can be performed regardless of ASD anatomy and may lead to a more effective and durable repair. MICS is associated with similar or even lower complication rates, including infections and bleeding, compared to median sternotomy, in addition to offering better cosmetic results [5].

Patch closure using the MICS approach is common in ASDs. However, to our knowledge, patch closure using the MICS approach for CS-ASD has not yet been reported. A literature search revealed no previous reports of patch closure for CS-ASD performed under thoracoscopic guidance, suggesting this may be the first documented case. Nonetheless, the possibility of unpublished or non-indexed cases cannot be excluded.

CS-ASD is commonly closed using the patch closure technique in the field of pediatric cardiac surgery, with a pericardial patch to redirect the CS drainage to the LA. In the present case, patch closure was performed without rerouting the CS to the LA, because the defect was small, localized, and not associated with a PLSVC. Rerouting was deemed unnecessary, and maintaining the native CS drainage to the RA minimized the risk of long-term stenosis or flow disturbance. Long-term CS patency was not considered a major concern due to the limited extent of the patch.

The key to patch closure in CS-ASD is to understand the conduction system and suture the patch to avoid conduction disturbances. The atrioventricular node in CS-ASD is at the apex of the Koch triangle, as expected in a normal heart. With good visualization through endoscopy, it is easy to determine the position of the atrioventricular node in MICS, and the CS-ASD can be closed using a patch that circumvents the conduction system.

An LV thrombus is treated primarily with anticoagulants; however, LV thrombectomy is an effective treatment that involves a high risk of systemic embolization. In this case, surgical removal was chosen after three months of appropriate anticoagulation therapy failed to achieve thrombus resolution. Given the mobile nature and embolic risk of the thrombus, surgery was deemed necessary despite the standard recommendation for longer anticoagulation in some cases.

Left ventriculotomy is a conventional approach to LV thrombosis [6], but transaortic and transmitral approaches have been described as substitutes for LV incision, which aids in maintaining LV function. These alternative approaches, including the transmitral route, avoid myocardial incision and thereby reduce the risk of postoperative LV dysfunction and scarring [7,8].

LV thrombectomy was performed under endoscopic guidance via a transmitral approach through a right mini-thoracotomy. This method is advantageous because it does not require an LV incision, provides clear surgical visualization using endoscopy, and preserves the sternum [9]. However, it is important to confirm the location of the thrombus preoperatively, as observation of the anterior LV wall is considered relatively difficult by endoscopy.

In this case, the thrombus was localized preoperatively using contrast-enhanced CT, which clearly demonstrated a mass attached to the inferolateral wall of the left ventricle. Intraoperative TEE was also used to confirm complete thrombus removal and assess for residual debris or wall motion abnormalities, compensating for the limitations of endoscopic visualization in anterior segments. If the thrombus is in the anterior wall or if there are multiple thrombi, a left ventriculotomy is more suitable for a thorough examination of the cavity.

This patient had CS-ASD with a coexisting LV thrombus. By combining fundamental MICS techniques, including endoscopic approaches, we were able to safely perform both patch closure and thrombectomy through small incisions. Although this approach may involve slightly longer cross-clamp or CPB times due to the intricacy of the techniques and need for thorough visualization, it provides excellent access and preserves ventricular function and the sternum. In our case, the operative times were within an acceptable range and did not lead to adverse outcomes. Endoscopic surgery, despite its many steps, can be readily and safely executed.

## Conclusions

We present a rare case of CS-ASD coexisting with LV thrombosis treated simultaneously through right mini-thoracotomy. To our knowledge, there have been no previous reports describing the coexistence of these two conditions. Although this is a procedure with many steps, it can be carried out with minimal complexity by combining basic MICS techniques, provided that the operator has sufficient experience in MICS, as the learning curve may be steep for beginners. This approach may facilitate earlier postoperative recovery compared to median sternotomy. Additionally, minimally invasive access may reduce the risk of wound complications and improve cosmetic outcomes. Long-term follow-up is essential to monitor the durability of the repair and evaluate the risk of recurrent thrombus formation.

## References

[1] Raghib G, Ruttenberg HD, Anderson RC, Amplatz K, Adams P Jr, Edwards JE (1965). Termination of left superior vena cava in left atrium, atrial septal defect, and absence of coronary sinus; a developmental complex. Circulation.

[2] Kouchoukos NT, Blackstone EH, Hanley FL, Kirklin JK (2013). Cardiac Surgery. 4th ed. Cardiac Surgery 4th ed. Philadelphia: Elsevier Saunders.

[3] Kijima Y, Taniguchi M, Akagi T (2012). Catheter closure of coronary sinus atrial septal defect using Amplatzer Septal Occluder. Cardiol Young.

[4] Mylonas KS, Ziogas IA, Evangeliou A (2020). Minimally invasive surgery vs device closure for atrial septal defects: a systematic review and meta-analysis. Pediatr Cardiol.

[5] Mishaly D, Ghosh P, Preisman S (2008). Minimally invasive congenital cardiac surgery through right anterior minithoracotomy approach. Ann Thorac Surg.

[6] Yadava OP, Yadav S, Juneja S, Chopra VK, Passey R, Ghadiok R (2003). Left ventricular thrombus sans overt cardiac pathology. Ann Thorac Surg.

[7] Tsukube T, Okada M, Ootaki Y, Tsuji Y, Yamashita C (1999). Transaortic video-assisted removal of a left ventricular thrombus. Ann Thorac Surg.

[8] Osada H, Nakajima H, Meshii K, Ohnaka M (2015). Transmitral, video-assisted left ventricular thrombectomy. Eur J Cardiothorac Surg.

[9] Koizumi S, Ishida K (2021). Endoscopy-assisted removal of multiple left ventricular thrombi through right mini-thoracotomy. Gen Thorac Cardiovasc Surg.

